# Mid-Upper Arm Circumference as a Surrogate for Nutritional Assessment in Surgical Patients: Insights from Global Leadership Initiative on Malnutrition Criteria Screening

**DOI:** 10.21315/mjms-10-2024-789

**Published:** 2025-02-28

**Authors:** Siang Poon Goh, Mawaddah Azman, Zaleha Md Isa

**Affiliations:** 1 Department of Otorhinolaryngology Head and Neck Surgery, Faculty of Medicine, Universiti Kebangsaan Malaysia Medical Centre, Kuala Lumpur, Malaysia; 2 Department of Public Health Medicine, Faculty of Medicine, Universiti Kebangsaan Malaysia Medical Centre, Kuala Lumpur, Malaysia

**Keywords:** BMI, malnutrition, obesity, fat-free mass index, overweight, MUAC

## Abstract

**Background:**

Undernutrition significantly affects surgical patient outcomes, prompting the Global Leadership Initiative on Malnutrition (GLIM) to establish a set of diagnostic criteria. This study assessed the prevalence of undernutrition using the GLIM criteria and determined a mid-upper arm circumference (MUAC) cutoff for malnutrition.

**Methods:**

This cross-sectional study was conducted from December 2021 to May 2023. Biodata, information necessary for GLIM criteria, and anthropometric measurements including height, body weight, MUAC, fat mass (FM), and fat-free mass (FFM), were collected. Correlations among indicators analysed using Pearson’s correlation. The MUAC cutoff points for underweight, obesity, and undernourishment were derived using receiver operating characteristics (ROC) and area under the curve (AUC), and the sensitivity, specificity, and positive and negative predictive values were calculated.

**Results:**

Despite a mean body mass index (BMI) of 25.8 (6.0) kg/m^2^, 30.0% of the patients met the GLIM undernutrition criteria. Overall, 13.4% of the patients were overweight, and 54.2% were obese. ROC analysis showed MUAC measures with an AUC ranging from 0.66–0.97. A MUAC cutoff of 28.9 cm identified undernourished patients with 66.7% sensitivity, 67.1% specificity, and 46.6% positive predictive value (PPV). Notably, the PPV increased to 75.4% in patients with cancer. The optimal MUAC cutoffs derived from the fat-free mass index (FFMI) for undernourished males and females were 26.8 cm and 26.1 cm, respectively.

**Conclusion:**

Malnutrition is prevalent among surgical patients. The MUAC is a promising surrogate for assessing BMI and FFMI and serves as a valuable screening tool for nutritional status. These findings emphasise the importance of nutritional assessment in surgical care.

## Introduction

Undernutrition and obesity are acknowledged prognostic indicators of postoperative complications, including infection, impaired wound healing, delayed recovery, prolonged hospitalisation, increased healthcare costs, and diminished quality of life (1–3). Undernutrition is linked to unfavourable oncological outcomes, whereas a high body mass index (BMI) is associated with elevated perioperative and postoperative morbidity rates (4). Consequently, there is an urgent need for rapid, easy, and cost-effective screening tools in busy clinics to promptly identify malnourished patients and initiate timely interventions.

The Global Leadership Initiative on Malnutrition (GLIM) has recently advocated a comprehensive diagnostic assessment of malnutrition incorporating a combination of phenotypic and etiologic criteria. The phenotypic criteria include weight loss, low BMI, and reduced fat-free mass index (FFMI), whereas the etiologic criteria include diminished food intake or assimilation and the presence of disease burden or inflammation. To establish a diagnosis of malnutrition, at least one phenotypic and etiologic criterion must be met. Patients diagnosed with malnutrition can be further classified into moderate or severe forms based on phenotypic criteria (5).

BMI is a widely utilised and extensively researched phenotypic criterion outlined by GLIM, featuring established cutoff points for underweight, overweight, and obesity. Nevertheless, specific medical conditions such as ascites, peripheral oedema, kyphosis, or scoliosis can lead to an increase in body weight and a decrease in height, thereby compromising the reliability of BMI values. Additionally, BMI does not assess body composition and tends to underestimate fat-free mass (FFM) depletion (6).

Bioelectrical impedance analysis (BIA) has emerged as a noninvasive, quick, and safe tool for nutritional measurements and has recently gained popularity. BIA operates on the principle that electrical current moves more rapidly through tissues with higher water and electrolyte contents than through less-hydrated tissues. FFM can be determined using various population-specific equations that have been developed and validated (6). However, it is important to note that certain conditions or diseases leading to severe changes in hydration status, extreme BMI, or the presence of prosthetics may impact the accuracy of BIA (7).

The mid-upper arm circumference (MUAC) is a noninvasive, swift, cost-effective, portable, and straightforward method for evaluating muscle mass in office-based settings. Its well-established status and widespread use as a screening tool for identifying malnutrition in children and women of reproductive age are noteworthy (6, 8, 9). Due to its robust correlation with BMI, MUAC has been proposed as a viable tool for nutritional screening (10).

Published literature on MUAC has not incorporated a combination of phenotypic and etiologic criteria for malnutrition screening. The authors express their confidence in the efficacy of the newly proposed GLIM criteria as a robust screening protocol for malnutrition in adults. Given the limitations of the BMI as an anthropometric measurement, and the limited accessibility of BIA in many clinical settings, there is a need to investigate the validity of alternative measures that are more clinically relevant.

This study aimed to determine the nutritional status of patients from various surgical disciplines in a tertiary educational centre using the GLIM criteria and various nutritional parameters. Additionally, we investigated the correlations between BMI, FFMI, and MUAC. Furthermore, the study investigated MUAC as a surrogate for BMI, FFMI, and GLIM criteria and endeavoured to establish cutoff circumferences indicative of adult malnutrition.

## Methods

### Study Design

This cross-sectional study was conducted in surgical wards and clinics at Hospital Canselor Tuanku Muhriz from December 2021 to May 2023, spanning a duration of 18 months.

### Sample Size

Sample size was calculated based on the estimated prevalence in the targeted population. This prevalence was extrapolated from the study of Jamhuri et al. (11), in which 62% of hospitalised patients were identified as undernourished. The margin of error was set within ± 5.0%. Therefore, 369 subjects were required. After adding a 10% non-response rate, the final sample size was 406. To assess the correlation between anthropometric parameters, a minimum sample of 84 participants was required to detect a coefficient of 0.3 with a 95% confidence interval (12).

Considering an estimated 30% prevalence of malnutrition among surgical patients, a minimum sample size of 103 participants is needed to achieve a minimum power of 80% for detecting a change in the sensitivity of a screening test from 0.7 to 0.9, based on a target significance level of 0.05 (12).

### Inclusion and Exclusion Criteria

The inclusion criterion was an age of at least 18 years old. Patients with and without nutritional support were recruited for this study. Most participants were in the preoperative phase, whereas a smaller percentage included postsurgical patients who had already passed the acute phase of recovery. The exclusion criteria were pregnancy, electrolyte imbalance, diuretic use, limb amputation, pacemaker implantation, recent trauma, or surgery affecting the arms.

### Ethical Approval

This study was approved by the University Kebangsaan Malaysia Ethics Review Board (ethics approval reference: UKM PPI/111/8/JEP-2021-786). The study adhered to the guidelines outlined in the Declaration of Helsinki. All participants provided informed consent.

### Data Collection

All participants were instructed to wear hospital gowns and remove all accessories and footwear. Subsequently, the participants were interviewed to obtain informed consent, biodata, and details pertaining to appetite, duration of poor appetite, previous weight, and underlying medical conditions and illnesses, which were extracted from medical case notes.

The participants’ heights were measured using a calibrated SECA scale (Seca GmbH, Hamburg, Germany). Body weight, fat mass (FM), FFM, and BMI were determined using a Tanita TBF-300A Total Body Composition Analyzer.

The MUAC was measured on the non-dominant upper limb of the participants with the arm flexed to 90 ° from the elbow. The mid-arm point was marked at the midpoint between the shoulder acromion and elbow olecranon processes. The participants allowed their non-dominant arm to hang loosely, and the investigator measured the area around the midpoint of the upper arm. Three MUAC measurements were recorded. All the data were documented using Google Forms (Google Inc., USA).

### Operational Definitions of Variables

MBI was classified based on Asian-Pacific BMI guidelines as follows: underweight (BMI < 18.5 kg/m^2^ for individuals aged < 70 years old; BMI < 20 kg/m^2^ for individuals aged ≥ 70 years old), normal (BMI < 23 kg/m^2^), overweight (BMI ≥ 23 kg/m^2^ and < 25 kg/m^2^), and obese (BMI ≥ 25 kg/m^2^) (13).

While there is no universally standardised FFMI value for assessing nutritional status, the authors of this study established criteria. For males, an FFMI value < 16.3 kg/m^2^ is defined as underweight, and for females, it was < 14.6 kg/m^2^. Conversely, an FFMI value exceeding 21.3 kg/m^2^ in males and 17.5 kg/m^2^ in females are indicative of obesity (14).

Participants who were classified into the undernutrition category met at least one of the phenotypic and etiologic criteria outlined by the GLIM. Phenotypic criteria consist of weight loss exceeding 5% within 6 months or more than 10% over more than 6 months, a low BMI (< 18.5 kg/m^2^ if aged < 70 years old or < 20 kg/m^2^ if aged ≥ 70 years old), or an FFMI < 16.3 kg/m^2^ in males and < 14.6 kg/m^2^ in females (5, 14). Etiological criteria include reduced food intake of more than 50% of energy requirements for over 1 week, any reduction for more than 2 weeks, or reduction in assimilation, such as in chronic gastrointestinal conditions adversely affecting food assimilation, or the presence of acute disease/injury or chronic disease.

### Statistical Analysis

Data were analysed using IBM SPSS version 29 (IBM Corp., Armonk, New, USA). Numerical variables were expressed as mean and standard deviation (SD), and categorical variables were expressed as frequencies and percentages. Correlations between BMI, MUAC, and FFMI were assessed using Pearson’s correlation. Specifically, the correlations between FFMI, BMI, and MUAC were examined within a BMI range of 15–32 kg/m^2^. The validity of the surrogate measures was established using receiver operating characteristic (ROC) analysis with metrics including area under the curve (AUC), sensitivity, specificity, Youden’s index (YI), positive predictive value (PPV), and negative predictive value (NPV). The MUAC cutoff points with the highest YI values were deemed optimal for statistically deriving the thresholds for undernourished and obese individuals.

## Results

### Demographic and Anthropometric Characteristics of Study Population

The study sample comprised of 439 patients (mean age, 47.5 (17.4) years old). The sex distribution in this study was relatively even, with 49.6% of the participants identified as male and 50.4% as female. Ethnically, 57.4% were Malay (*n* = 252), 30.1% were Chinese (*n* = 132), 9.8% were Indian (*n* = 43), and 2.7% were of other ethnicities (*n* = 43). The surgical patients in the study had a mean BMI of 25.8 (6.0) kg/m^2^, a mean FFMI of 16.8 (2.6) kg/m^2^, and a mean MUAC of 29.9 (1.1) cm. Additional anthropometric measurements are shown in Table 1.

### Nutritional Status of Surgical Patients

According to the GLIM criteria, 30.1% (*n* = 132) of the surgical patients were classified as undernourished, while 69.9% (*n* = 307) were deemed well-nourished. The results of the nutritional status indicators, including BMI, FFMI, and MUAC, are summarised in Table 1.

The distribution of the samples according to the surgical discipline was as follows: otorhinolaryngology, 52.2% (*n* = 230); surgery, 33.6% (*n* = 147); oromaxillofacial surgery, 6.9% (*n* =30); ophthalmology, 2.7% (*n* = 12); plastic surgery, 1.8% (*n* = 8); orthopaedic surgery, 1.8% (*n* = 8); and neurosurgery, 1% (*n* = 4). No significant differences in nutritional status were observed between the various surgical disciplines (Table 2).

Among patients with cancer, 70 (57.4%) met the criteria for GLIM-defined malnutrition, signifying a significant association between cancer and malnutrition risk (Table 2).

### Correlations Among Various Anthropometric Measurements

Following Cohen’s guidelines for correlation (*r* = 0.1, small; *r* = 0.3, moderate; and *r* = 0.5, strong), MUAC demonstrated strong correlations with BMI (*r* = 0.939) and male FFMI (*r* = 0.547), and a moderate correlation with female FFMI (*r* = 0.365). BMI strongly correlated with FM (*r* = 0.906). However, BMI showed varying strengths of correlation with FFM and FFMI in the different sexes. A summary of the results is presented in Table 3.

### MUAC Cutoff Points for Nutritional Status

Given that FFMI is not highly sensitive to obesity, MUAC cutoff points derived from BMI were identified, with 24.4 cm for underweight and 29.8 cm for obesity. These cutoff points exhibited high sensitivity (> 0.85). MUAC cutoff points derived from mixed-sex FFMI and female FFMI were 26.7 cm and 26.1 cm, respectively, both demonstrating relatively lower sensitivities and YIs. The MUAC cutoff point derived from male FFMI was 26.8 cm, displaying a relatively higher sensitivity (78.3%). The MUAC cutoff value derived from the GLIM criteria was 28.9 cm, with an AUC of 0.694, sensitivity of 69.4%, and specificity of 67.1%. A summary of the various MUAC cutoff points for nutritional status is presented in Table 4.

### MUAC as the Surrogates for BMI and FFMI

In identifying undernourished patients, the MUAC cutoff point derived from sex-specific FFMI exhibited the highest PPV of 65.3% and an NPV of 82.5%. Conversely, the PPV of MUAC derived from BMI was 52.2%, but it had the highest NPV of 99.7%, surpassing that of MUAC derived from FFMI. To detect obesity, the MUAC cutoff point derived from BMI demonstrated a high PPV of 94.9% and a high NPV of 85.6%. Table 5 summarises the PPVs and NPVs for the MUAC cutoff points.

The Spearman’s correlation coefficient for the MUAC and GLIM criteria was 0.309 (*p* < 0.001). The MUAC cutoff point for detecting undernourished patients was determined to be 28.9 cm, demonstrating a sensitivity of 66.7% and specificity of 67.1%. The PPV of this MUAC cutoff point was 46.6%, and the NPV was 82.4%. Remarkably, the PPV increased to 75.4% when specifically applied to patients with cancer.

## Discussion

### Nutritional Status of Surgical Patients

According to the GLIM-defined undernutrition criteria, this study revealed a 30.1% prevalence of undernourished patients among the overall surgical patient population, reaching 57.4% among those with surgical cancer. Notably, our study’s undernutrition prevalence among patients with cancer significantly surpassed that reported in a recent meta-analysis by Matsui et al. (ranging from 30%–40%) (3). Furthermore, local data from the Malaysia Cancer Institute, employing the Subjective Global Assessment, indicated that 43.5% of patients with cancer were malnourished (15).

The transition from underweight to overweight and obese has occurred rapidly globally over the past half-decade (16). This trend is evident in Malaysia, with recent prevalence rates of overweight and obesity of 30.4% and 19.7%, respectively (17). In the context of our study, the mean BMI of surgical patients was 25.8 (6.0) kg/m^2^. The prevalence of overweight and obesity among the surgical patients was 13.4% and 54.2%, respectively, based on BMI. This underscores the importance of a thorough assessment to identify undernourished individuals among surgical patients with a higher BMI.

The present data highlight the burden of both undernutrition and obesity on the healthcare system. Therefore, malnutrition should not be ignored in patients undergoing surgery. These findings emphasise the need for clinicians to remain vigilant in identifying undernourished and obese patients early, to intervene in a timely manner for better clinical outcomes.

### Comparison of Nutritional Status of the Surgical Patients using BMI, FFMI and MUAC

The data revealed that when BMI was used alone, only 37 (8.4%) surgical patients fell into the underweight category. However, when employing FFMI, the number of undernourished patients increased substantially, reaching 136 (31.0%), more than four times larger than the BMI-defined underweight category. Moreover, the number of undernourished patients defined by FFMI closely aligned with GLIM-defined undernutrition (*n* = 132, 30.1%). This underscores the greater sensitivity of FFMI in identifying undernourished individuals compared with BMI alone.

While FFMI may not be an ideal tool for assessing the morbidly obese population (BMI > 34 kg/m^2^) owing to potential interference effects such as abnormal hydration, increased fat fraction, and abnormal geometric tissue distribution in obese individuals (7), it still holds relevance with cautious interpretation. The FFMI is particularly meaningful when it identifies patients with obesity within the undernourished category. In contrast, BMI may not effectively indicate undernourishment in the obese population, as significant weight loss is required before an individual with obesity can be classified as underweight based on BMI alone. For the same reason, the percentage weight loss is listed as a GLIM phenotypic criterion. Therefore, BMI is not reliable for revealing undernourishment in obese populations. However, BMI remains a useful indicator for identifying overweight status and assessing the severity of obesity, especially in individuals who do not fall within the FFMI-defined- or GLIM-defined undernourished groups.

The substantial proportional difference in normal-to-obesity, as indicated by the BMI-defined- and FFMI-defined categories in Table 4, can be attributed to several factors. First, FFMI is effective in detecting low lean body mass in individuals with both normal and high BMI. Second, the reference used for the FFMI-defined normal BMI included individuals with both normal and overweight BMI. Finally, as mentioned earlier, FFMI may not accurately reflect the body composition in the obese population, thus contributing to the observed differences.

Recent studies have proposed a MUAC cutoff of approximately 23.5 cm to define undernourishment (10, 18). However, when applying MUAC 23.5 cm in our study, it only identified 11% of cases, failing to detect more than half of the patients who were GLIM-defined as undernourished. This suggests the need for a new MUAC cutoff point as a screening tool.

### MUAC as Surrogate Measures of BMI and FFMI

In busy outpatient clinics or rural areas, BMI is not routinely measured unless specifically ordered by clinicians. Additionally, BIA devices for FFMI measurements are expensive and unavailable. The process of removing shoes, accessories, and items from pockets is time consuming and poses a burden to understaffed clinics. Therefore, the use of MUAC as a surrogate for BMI and FFMI has been extensively investigated in both children and adults and has proven promising (10, 18–22). The ultimate goal of using the MUAC as a surrogate is to use it as a screening tool for nutritional status.

In comparison to previous studies and proposed MUAC values, whether as a surrogate for BMI or FFMI, our suggested sex-specific MUAC cutoff values for the undernourished group are relatively high (MUAC ≤ 26.8 cm for males and MUAC ≤ 26.1 cm for females). These MUAC values resulted in a sensitivity of 59.6% and a specificity of 85.8%. They exhibited a PPV of 65.3% and an NPV of 82.5%.

As shown in Table 5, the MUAC ≤ 23.5 cm cutoff proposed by previous studies, which is close to the values reported in the literature, demonstrated the highest PPV at 90.4%. However, it has a relatively low sensitivity (34.6 %). This suggests that using a lower MUAC cutoff may result in a significant number of undernourished patients being missed.

This study proposes a MUAC cutoff value of ≥ 29.8 cm as a screening tool for obesity. This cutoff MUAC value exhibited high sensitivity (86.6%) and specificity (94.5%). It is noteworthy that the proposed MUAC cutoff value aligns closely with a large sample size study of obese individuals in the Asian population, where 2,427 individuals with a mean BMI of 25.5 (2.61) kg/m^2^ had a mean MUAC of 29.6 (0.53) cm, and 2,427 individuals with a mean BMI of 26.9 (3.1) kg/m^2^ had a mean MUAC of 32.5 (1.7) cm (23). However, it is important to note that this MUAC cutoff value may not be applicable to athletes or bodybuilders, as they typically have a higher lean body mass and lower FM.

### Cutoff MUAC Values Derived from GLIM for Undernutrition

To the best of our knowledge, this study is the first to propose a MUAC cutoff value (MUAC ≤ 28.9 cm) to predict undernourished patients. Its sensitivity and PPV increased notably when applied to patients with a positive aetiology, making a clinically relevant screening tool. This is because the MUAC cutoff is directly derived from the GLIM criteria, offering a more straightforward approach than assessing multiple phenotypes, such as BMI, FFMI, and percentage of weight loss. As discussed earlier, issues with BMI and FFMI include potential inaccuracies, and in some cases, patients may not have sufficient information about their previous body weight to quantify weight loss over time. While the GLIM criteria have demonstrated their usefulness, it is acknowledged that not all clinicians are up-to-date or remember each phenotype and aetiology criterion. In such situations, a single MUAC, which is less time consuming to measure, is a desirable screening tool.

### Limitations

This study had several limitations. First, the participants were recruited from various surgical disciplines, and the distribution of participants across subsurgical disciplines was uneven. Different surgical diseases can have diverse impacts on patients’ nutritional status; therefore, the prevalence of malnutrition may not be representative of every subsurgical department. Second, the study included a small number of subjects with a very high BMI (n=35, BMI > 34 kg/m^2^), which could potentially impact the mean values of general anthropometric parameters. However, subjects with extreme BMI were excluded from the FFMI-related analyses. Third, there was a lack of information regarding the Asian FFMI-defined nutritional status. The reference FFMI values used in this study were obtained from a European population derived from BMI (14). Consequently, this study did not include an overweight group in the FFMI-defined nutritional status, raising questions about the prevalence of FFMI-defined normal-to-obesity. Fourth, the sample size of this study was relatively small compared with that of high-impact publications (21). Fifth, other muscle parameters such as handgrip strength and gait speed have been shown to be reliable predictors of nutritional status and clinical outcomes. However, we did not have access to the devices required to measure these parameters. Further studies that incorporate muscle parameters are highly recommended.

## Conclusion

The prevalence of malnutrition in surgical patients, including both undernourished and obese individuals, was notably high. Approximately 30.0% of the patients met the criteria for GLIM-defined undernutrition, whereas approximately 54.0% were classified as having BMI-defined obesity. MUAC demonstrated potential as a surrogate for BMI and FFMI in various applications. Patients with a MUAC of ≤ 28.9 cm should be considered for further investigation for malnutrition and early intervention as clinically indicated. Establishing an Asian FFMI-defined nutritional status as a reference and guide for future studies is crucial.

## Figures and Tables

**Table 1:** Demographics and anthropometric characteristics of the study population (*n* = 439) and the prevalence of different nutritional statuses

Demographics		*n* (%)	Mean (SD)
**Age Group (year)**	20	15 (3.4)	
	21–40	168 (38.3)	
	41–60	121 (27.6)	
	> 60	135 (30.7)	

**Sex**	Male	218 (49.6)	
	Female	221 (50.4)	

**Ethnicity**	Malay	252 (57.4)	
	Chinese	132 (30.1)	
	Indian	43 (9.8)	
	Others	12 (2.7)	

**Anthropometrics parameters**	Weight, kg		67.9 (18.4)
	Height, m		1.62 (0.1)
	BMI, kg/m^2^		25.8 (6.0)
	FM, kg		23.6 (14.2)
	FFM, kg		44.4 (9.9)
	FFMI, kg/m^2^		16.8 (2.6)
	MUAC, cm		29.9 (1.1)

**Nutritional status**	GLIM		
	Undernourished	132 (30.1)	
	Well-nourished	307 (69.9)	
	BMI		
	Underweight	37 (8.4)	
	Normal	105 (24.0)	
	Overweight	59 (13.4)	
	Obesity	238 (54.2)	
	FFMI		
	Undernourished	136 (31.0)	
	Normal	263 (59.9)	
	Obesity	40 (9.1)	

Notes: BMI = body mass index; FFM = fat-free mass; FFMI = fat-free mass index; FM = fat mass; GLIM = Global Leadership Initiative on Malnutrition; MUAC = mid-upper arm circumference; SD = standard deviation

**Table 2:** Prevalence of GLIM-malnourished patients across different surgical disciplines and in patients with cancer

Variables	Participant	GLIM-malnourished		*P*-value^a^

	*n* (%)	*n*	% (95% CI)	
**Disciplines**				0.206
Otorhinolaryngology	230 (52.2)	70	30.4 (24.8–6.6)	
Surgery	147 (33.6)	51	34.7 (27.4–42.6)	
Oro-maxillofacial surgery	30 (6.9)	7	23.3 (11.1–40.4)	
Ophthalmology	12 (2.7)	1	8.3 0.9–32.8)	
Orthopaedics	8 (1.8)	0	0 (-)	
Plastics Surgery	8 (1.8)	2	25 (5.6–59.2)	
Neurosurgery	4 (1.0)	1	25.0 (2.8–42.6)	

**Cancer**				< 0.001
Yes	122 (27.8)	70	57.4 (48.5–65.9)	
No	317 (72.2)	62	19.6 (15.5–4.2)	

Notes:

a Chi square test;

BMI = body mass index; CI = confidence interval; FFM = fat-free mass; FFMI = fat-free mass index; FM = fat mass; GLIM = Global Leadership Initiative on Malnutrition; MUAC = mid-upper arm circumference

**Table 3:** Correlations among BMI, FM, FFM, FFMI and MUAC (*n* = 439)

Variable 1	Variable 2	Coefficient of correlation, *r*	*P*-value^a^
MUAC	BMI	0.939	< 0.001
	Non sex-specific FFMI	0.429	< 0.001
	Male FFMI	0.547	< 0.001
	Female FFMI	0.365	< 0.001

BMI (overall)	FM	0.906	< 0.001
	FFM	0.361	< 0.001
	FFMI	0.463	< 0.001

BMI (male)	FM	0.814	< 0.001
	FFM	0.669	< 0.001
	FFMI	0.735	< 0.001

BMI (female)	FM	0.924	< 0.001
	FFM	0.326	< 0.001
	FFMI	0.456	< 0.001

Notes:

a Pearson correlation;

BMI = body mass index; FFMI = fat-free mass index; MUAC = mid-upper arm circumference.

**Table 4:** MUAC cutoff points for nutritional status

Variable	Nutritional status	MUAC cutoff point, cm	AUC (95% CI)	Sensitivity (%)	Specificity (%)
BMI	Underweight	24.4	0.977 (0.963–0.992)	97.3	91.8
	Obesity	29.8	0.965 (0.951–0.979)	86.6	94.5

Non sex-specific FFMI	Undernourished	26.7	0.739 (0.682–0.796)	65.8	81.4

Male FFMI	Undernourished	26.8	0.875 (0.804–0.945)	78.3	88.7

Female FFMI	Undernourished	26.1	0.667 (0.581–0.753)	54.4	76.7

GLIM	Undernourished	28.9	0.694 (0.64–0.749)	66.7	67.1

Notes: AUC = area under the curve; BMI = body mass index; CI = confidence interval; FFMI = fat-free mass index; GLIM = Global Leadership Initiative on Malnutrition; MUAC = mid-upper arm circumference

**Table 5:** Predictive values of MUAC as surrogates to detect malnutrition status in surgical patients

Variable	Nutritional status, MUAC (cm)	Sensitivity (%)	Specificity (%)	PPV (%)	NPV (%)	*P*-value^a^
BMI	Underweight, MUAC 24.4	97.3	91.8	52.2	99.7	< 0.001
	Obesity, MUAC 29.8	86.6	94.5	94.9	85.6	< 0.001

Non sex-specific FFMI	Undernourished, MUAC 26.7	65.8	81.4	56.7	82.4	< 0.001

Sex-specific FFMI	Undernourished, (male) MUAC 26.8; (female) MUAC 26.1	59.6	85.8	65.3	82.5	< 0.001

Previous study FFMI	Undernourished, MUAC 23.5	34.8	98.3	90.4	77.0	< 0.001

GLIM	Undernourished, MUAC 28.9	66.7	67.1	46.6	82.4	< 0.001

GLIM in patients with cancer	Undernourished, MUAC 28.9	71.4	67.3	75.4	66.0	< 0.001

Notes:

a Pearson chi-square test;

BMI = body mass index; FFMI = fat-free mass index; GLIM = Global Leadership Initiative on Malnutrition; MUAC = mid-upper arm circumference; NPV = negative predictive value; PPV = positive predictive value
